# Electroformation of Giant Unilamellar Vesicles from Damp Films in Conditions Involving High Cholesterol Contents, Charged Lipids, and Saline Solutions

**DOI:** 10.3390/membranes14100215

**Published:** 2024-10-12

**Authors:** Ivan Mardešić, Zvonimir Boban, Marija Raguz

**Affiliations:** 1 Department of Medical Physics and Biophysics, University of Split School of Medicine, 21000 Split, Croatia; ivan.mardesic@mefst.hr (I.M.); zvonimir.boban@mefst.hr (Z.B.); 2 Doctoral Study of Biophysics, Faculty of Science, University of Split, 21000 Split, Croatia

**Keywords:** GUV, electroformation, damp lipid film, cholesterol demixing artifact, cholesterol, charged lipids, saline solution, lipid film deposition

## Abstract

Giant unilamellar vesicles (GUVs) are frequently used as membrane models in studies of membrane properties. They are most often produced using the electroformation method. However, there are a number of parameters that can influence the success of the procedure. Some of the most common conditions that have been shown to have a negative effect on GUV electroformation are the presence of high cholesterol (Chol) concentrations, the use of mixtures containing charged lipids, and the solutions with an elevated ionic strength. High Chol concentrations are problematic for the traditional electroformation protocol as it involves the formation of a dry lipid film by complete evaporation of the organic solvent from the lipid mixture. During drying, anhydrous Chol crystals form. They are not involved in the formation of the lipid bilayer, resulting in a lower Chol concentration in the vesicle bilayer compared to the original lipid mixture. Motivated primarily by the issue of artifactual Chol demixing, we have modified the electroformation protocol by incorporating the techniques of rapid solvent exchange (RSE), ultrasonication, plasma cleaning, and spin-coating for reproducible production of GUVs from damp lipid films. Aside from decreasing Chol demixing, we have shown that the method can also be used to produce GUVs from lipid mixtures with charged lipids and in ionic solutions used as internal solutions. A high yield of GUVs was obtained for Chol/1-palmitoyl-2-oleoyl-sn-glycero-3-phosphocholine (POPC) samples with mixing ratios ranging from 0 to 2.5. We also succeeded in preparing GUVs from mixtures containing up to 60 mol% of the charged lipid 1-palmitoyl-2-oleoyl-sn-glycero-3-phospho-L-serine (POPS) and in NaCl solutions with low ionic strength (<25 mM).

## 1. Introduction

Vesicles serve as vital tools for researchers exploring the membrane properties under controlled conditions. Based on their lamellarity, they are divided into unilamellar, multilamellar (MLVs), and oligolamellar vesicles. Unilamellar vesicles consist of a single lipid bilayer, multilamellar vesicles have several concentric lipid bilayers, and oligolamellar vesicles contain smaller vesicles within the outer membrane. Unilamellar vesicles are further subdivided according to their size into small (SUVs, diameter less than 100 nm), large (LUVs, diameter from 100 nm to 1 µm), and giant unilamellar vesicles (GUVs, diameter over 1 µm). While SUVs and LUVs are frequently used for drug delivery investigations, GUVs attract more attention for studies on membrane properties and organization, mainly due to their size, which is comparable to that of eukaryotic cells [1]. Furthermore, due to their size, GUVs can be observed using light microscopy techniques, which is an additional advantage for researchers [2].

In modern research, electroformation has become one of the most widely used methods for the production of GUVs [3]. In this technique, an electric field is applied to an electrode containing lipids to generate vesicles [4]. Typically, lipids dissolved in an organic solvent are deposited onto an electrode. The solvent is then evaporated and vacuum treatment is applied to remove the residual solvent, leaving a dry lipid film. The lipid-coated and lipid-free electrodes were used to assemble the electroformation chamber, which was then filled with a chosen internal solution and connected to an alternating current generator. The hydration of the lipid film, assisted by the electric field, promotes the detachment of the lipids, which leads to the formation of vesicles.

Electroformation offers significant advantages over the natural swelling method, including a higher yield of unilamellar vesicles and less heterogeneity in composition. However, the method is not without its challenges. The traditional electroformation protocol, which involves depositing lipids using the drop-deposition method, often results in lipid films with non-uniform thickness, leading to GUVs with broad size distribution and varying compositions, ultimately reducing experimental reproducibility.

Researchers have made various attempts to improve this process [5,6,7]. For instance, the spin-coating technique, in which a lipid solution is deposited onto a flat glass surface coated with indium tin oxide (ITO) and spun rapidly, has been employed to create uniform lipid films [8]. This method has been validated using techniques such as ellipsometry and atomic force microscopy and has gained acceptance among researchers employing a wide range of lipid compositions for GUV production [8,9,10,11,12,13,14].

**Figure 1:**
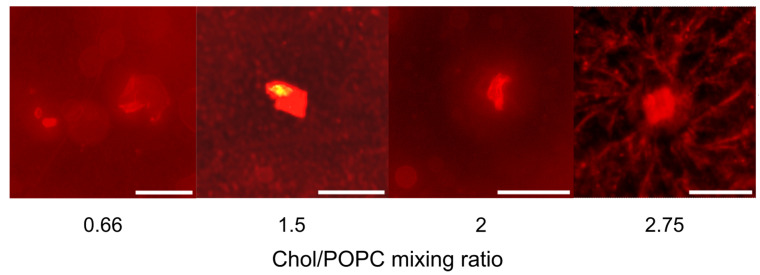
Examples of Chol crystals formed during the dry film phase when using the traditional electroformation protocol. The observed crystal structures are in good agreement with those observed in the study of Park et al. [21] on the phases of Chol crystallization. The scale bar represents 50 µm.

Another significant issue with the traditional protocol is cholesterol (Chol) demixing during the drying of the lipid film, particularly when working with lipid mixtures containing high Chol concentrations. In such cases, Chol can separate and form anhydrous Chol crystals, which do not participate in the formation of the lipid bilayer during rehydration, leading to a lower Chol concentration in the resulting bilayer (Figure 1) [9,15,16,17]. To address this issue, some researchers have utilized the rapid solvent exchange (RSE) technique, in which the drying phase is avoided by combining lipids dissolved in organic solvent with an aqueous solution, followed by the rapid evaporation of the organic solvent [18,19,20]. This method is effective in preventing Chol demixing; it primarily produces MLVs.

Recent advances in electroformation protocols have also focused on the cleaning of electrodes prior to lipid film deposition. Traditionally, electrodes are cleaned using organic solvents, but plasma cleaning has proven to be a more effective alternative. Plasma treatment not only cleans the electrodes but also enhances the efficiency of GUV formation, particularly when using buffers with physiological levels of charged particles, likely due to improved hydration of the lipid film [22,23].

In this study, we combine the above modifications with the traditional method of producing GUVs using an electroformation protocol from damp lipid films. This method has the potential to prevent Chol demixing during electrofromation while keeping the preparation time short and enhancing the reproducibility of the protocol. Such GUVs are of particular interest to researchers studying the role of Chol in various biological membranes, including those of the eye lens fiber cells, and in the development of atherosclerosis [20,24,25]. We show that the method is not only suitable to be applied to lipid mixtures with high Chol concentrations but can also be used to produce GUVs from mixtures with charged lipids or in ion-containing solutions.

## 2. Materials and Methods

### 2.1. Materials

1-palmitoyl-2-oleoyl-sn-glycero-3-phosphocholine (POPC), 1-palmitoyl-2-oleoyl-sn-glycero-3-phospho-L-serine (POPS), and Chol were purchased from Avanti Polar Lipids Inc. (Alabaster, AL, USA). The fluorescent dye 1,1-dioctadecyl-3,3,3,3-tetramethylindocarbocyanine Perchlorate (DiIC_18_(3)) was purchased from Invitrogen, Thermo Fisher Scientific (Waltham, MA, USA). Lipids were stored at −20 °C when not in use. ITO-coated glass (ICG-90 INS 115, resistance 70–100 Ω and dimensions of 25 × 75 × 1.1 mm) was purchased from Delta Technologies (Loveland, LO, USA). A fresh ITO-coated glass was used for each preparation to ensure efficient GUV formation [26]. Mili-Q deionized water (Merck, Rahway, NJ, USA), preheated to 60 °C, was used as the internal chamber solution. For the saline solutions, NaCl 99.5% (Gram-mol, Zagreb, Croatia) was dissolved in Mili-Q deionized water.

### 2.2. Preparation of Multilamellar Vesicles Using the Rapid Solvent Exchange Method

MLVs were produced using a custom-built rapid RSE device to resolve the issue of Chol demixing. A lipid mixture dissolved in chloroform was prepared by mixing 25 mg/mL of POPC, 25 mg/mL of Chol, 10 mg/mL of POPS, and 1 mg/mL of DiIC_18_(3) in the appropriate proportions. The Chol/POPC mixing ratios ranged from 0 to 2.5 (equivalent to a cholesterol mixing concentration of 0 to 71.4 mol%), while the DiIC_18_(3)/POPC mixing ratio was kept constant at 1/500. When working with POPS/POPC/Chol ternary mixtures, the Chol mixing concentration was kept at 10 mol%, and the POPS mixing concentration ranged from 5 to 60 mol%. The total lipid mass per sample was 2.1 mg. Either Mili-Q water or a NaCl solution with low ionic strength (10 and 25 mM) were used as internal solutions. In total, 400 µL of the internal solution of choice was added to the lipids dissolved in the organic solvent, and the resulting mixture was vortexed (Vortex IR, Star Lab, Blakelands, UK) at a speed of 2200 rpm. While vortexing, the pressure was kept at approximately 0.05 bar using a vacuum pump (HiScroll 6, Pfeiffer Vacuum, Asslar, Germany). The sample was maintained at this pressure for 90 s to ensure the full removal of any remaining organic solvent.

### 2.3. Preparation of Unilamellar Vesicles by Sonication

The MLV solution prepared by the RSE method was sonicated using an ultrasonic homogenizer (SONOPLUS Ultrasonic Homogenizer HD 4050, Bandelin, Berlin, Germany) and an ultrasonic tip (Probe TS 102, Bandelin, Berlin, Germany) connected at an ultrasonic converter (UW 50, Bandelin, Berlin, Germany). If not stated differently, the sonication lasted 30 min at an amplitude of 60% (81 µm). Pulsed sonication was used (0.5 s on, 0.5 s off) to allow cooling and reduce the risk of overheating. To keep the lipids above their mixing temperature, the glass vial with the vesicle solution was heated at 60 °C using a hotplate (Cimarec + stirring hotplate, Thermo Fisher Scientific, Waltham, MA, USA).

### 2.4. Preparation of the Damp Lipid Film

The ITO-coated glass stored in Mili-Q deionized water overnight was taken out and then wiped five times with lint-free cloths saturated with 70% ethanol. Afterward, the electrode underwent a 1-min oxygen plasma treatment using a plasma cleaner (PDC-002-HPCE with PLASMAFLO PDC-FMG-2 attachment, Harrick Plasma, Ithaca, NY, USA) connected to a vacuum pump (HiScroll 6, Pfeiffer Vacuum, Asslar, Germany). Subsequently, 550 µL of a LUV suspension was applied to the hydrophilic plasma-treated ITO-coated glass electrode and spun using a spin-coater (SM-150, Sawatec, Sax, Switzerland) to obtain the lipid film. The electrode was spun at 600 rpm, reaching full speed within 1 s. The spin-coating was performed for 30 s to ensure the formation of a uniform damp lipid film. To prevent unintended evaporation, the coated ITO-coated glass was immediately placed into a Petri dish and used to assemble an electroformation chamber.

### 2.5. Electroformation Protocol 

The electroformation chamber was assembled by sandwiching two ITO-coated glass slides (25 × 37.5 mm) with a 1.6 mm thick polytetrafluoroethylene (Teflon) spacer in between. The electrodes were made by cutting a 25 × 75 mm ITO-coated glass slide in half with a diamond pen cutter. To assemble the chamber, the lipid-coated glass was sealed to the Teflon spacer using vacuum grease, while the lipid-free electrode was sealed to the Teflon spacer on the opposite side. After the addition of 280 µL of Mili-Q water or saline solution, the stopper was sealed with vacuum grease. The care was taken that the grease did not come into contact with the water in order to prevent contamination that could affect GUV formation. The chamber was secured with clamps at three points along the electrodes—two near the stopper and one on the opposite side. The chamber was connected to a pulse generator (PSG 9080, Joy-IT, Neukirchen-Vluyn, Germany) and placed in an incubator set at 60 °C. Copper tape was applied to the outer edges of the electrodes to improve the wire–electrode contact. As in previous experiments with aqueous solutions, a voltage of 2 V and a frequency of 10 Hz were used [7,9,10,12]. In experiments using saline solution as the internal solution, a voltage of 4 V and a frequency of 500 Hz were applied. After 2 h, the pulse generator was turned off and the chamber was kept in the incubator for an hour.

### 2.6. Dynamic Light Scattering

Dynamic light scattering (DLS) was utilized to determine the hydrodynamic diameter and polydispersity index of the MLV and LUV suspensions (Litesizer 500, Anton Paar, Graz, Austria).

### 2.7. Fluorescence Imaging and Data Analysis

In order to comprehensively analyze the entire chamber volume, images were captured at 16 different regions of the sample. A total of 300 vesicles were randomly tracked from fluorescent images. If an image contained less than 300 vesicles, all visible vesicles were selected. To avoid bias, a subset of 100 vesicles was randomly chosen from these 300 vesicles for further analysis. Imaging was performed with a fluorescence microscope (Olympus BX51, Olympus, Tokyo, Japan), and vesicle diameters were measured using the line tool in Fiji software (ImageJ 1.53c) [27]. The numerical results are presented as the mean ± standard errors. Both data analysis and visualization were conducted using the R programming language [28].

## 3. Results and Discussion

### 3.1. The Protocol

The modifications of the protocol were inspired by the vesicle fusion method often used for the production of supported lipid bilayers [29,30,31,32,33]. In this method, an aqueous suspension of LUVs or SUVs is deposited on a hydrophilized surface. The hydrophilic surface causes vesicle rupturing, resulting in planar bilayers. Since our main motivation was to prevent Chol demixing induced during the drying of the lipid film, in order to utilize this new protocol, we first had to find a way to reliably create LUVs containing high Chol concentrations.

**Figure 2:**
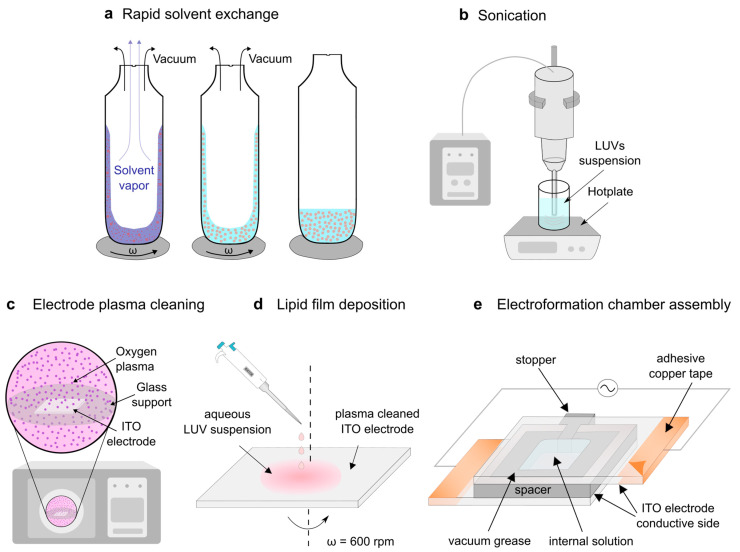
The modified protocol is for electroformation of GUVs from a damp lipid film. (**a**) The RSE method is used to produce an MLV solution. The RSE method is used to produce an MLV solution. An organic solvent (blue) containing lipids (red) is mixed with an aqueous solution (tube 1). The organic solvent is removed by vortexing the solution under vacuum in order to form MLVs (tube 3). (**b**) The suspension of MLVs is sonicated to produce LUVs. (**c**) A plasma cleaner is used to hydrophilize the ITO electrode. (**d**) The LUV suspension is deposited onto a plasma-cleaned ITO-coated glass and spin-coated to obtain a damp lipid film. (**e**) The coated electrode is used to assemble the electroformation chamber and connected to a voltage source to enable the growth of GUVs.

The easiest way to create vesicles is to use the hydration method. Unfortunately, this method also requires the evaporation of the organic solvent before the hydration of the film [34,35,36]. This is why our protocol begins with the preparation of MLVs using the RSE method (Figure 2a), which was confirmed to be effective in preventing Chol demixing [18,19]. The lipids dissolved in an organic solvent are first mixed with an aqueous solution of choice and stirred using a vortex mixer. The mixture is then subjected to a sudden vacuum, causing the organic solvent to rapidly evaporate from the mixture. 

Although effective, the RSE method does not provide a homogeneous suspension of LUVs, but instead contains MLVs instead. The preparation of LUVs from a suspension of MLVs is most commonly conducted using mechanical extrusion or sonication. We tested the extrusion approach in our previous work [10,12] but found a couple of drawbacks to the method. Firstly, the method is time-consuming and always results in a loss of a small portion of the suspension volume [37]. Secondly, the extrusion of MLVs with a high Chol content results in a very large opposing force, making the extrusion process difficult, especially when using smaller pore sizes [10,12].

For this reason, we have opted for the sonication technique in this study (Figure 2b). It is much simpler and faster than extrusion [37], with only one manual action required: submerging a sonicator tip into the suspension of MLVs. It is also cheaper in the long term, as there is no need to buy disposable filters, as is the case with extrusion.

After the LUV suspension was obtained, it should be deposited onto a hydrophilized electrode surface. The hydrophilization was achieved using a plasma cleaner (Figure 2c). The traditional way of lipid deposition is the drop deposition technique, but it leads to the creation of inhomogenous lipid films, reducing the reproducibility of the experiment [7]. Therefore, we opted to deposit the suspension of LUVs using the spin-coating method (Figure 2d), which was proven effective in creating homogeneous lipid films [8,10]. Additionally, by controlling the spin-coating duration, we can reproducibly control how wet the film is and prevent it from drying out completely [12].

Now that the electrode was coated with the damp lipid film, all that remained was to create an electroformation chamber and attach it to an alternating voltage source to facilitate the growth of the GUVs (Figure 2e).

### 3.2. Determination of the Sonication Parameters

**Figure 3:**
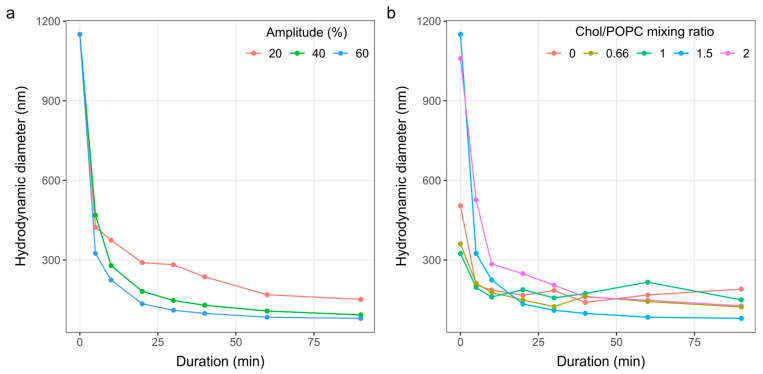
The effect of sonication parameters on the size of produced LUVs. (**a**) The effect of sonication duration using different sonication amplitudes for MLVs produced from a mixture with a Chol/POPC ratio of 1.5. (**b**) The effect of sonication duration for different Chol/POPC mixing ratios at a sonication amplitude of 60%.

The size distribution of vesicles can be influenced by factors such as lipid composition, sonication duration, and sonication amplitude [37,38,39]. Therefore, we first tested the effect of three different sonication amplitudes at a fixed Chol/POPC ratio of 1.5 (Figure 3a). The tested amplitudes were 20, 40, and 60%, with the amplitude of 100% corresponding to an oscillation of 135 µm. The amplitude was not increased above 60% in order to avoid possible excessive damage to the vesicles. In addition, pulsed sonication (0.5 s on, 0.5 s off) was utilized to allow cooling and reduce the risk of overheating. By continuously measuring the temperature throughout the duration of sonication, we did not detect any significant increase in temperature with this approach. After inspecting the rate of decrease in average vesicle size, we decided to use an amplitude of 60%.

Next, fixing the sonication amplitude at 60%, the effect of different lipid compositions on the change in particle size over time was tested (Figure 3b). Five different compositions were tested, with a Chol/POPC ratio ranging from 0 to 2. Similar to another study investigating the influence of increasing Chol concentration in the sonicated vesicles [38], we observed that vesicles with a lower Chol content broke more easily. However, after approximately 30 min, there seemed to be no appreciable additional decrease in average diameter with time. This saturation behavior is consistent with other studies on liposome sonication [39,40]. Furthermore, prolonged sonication induces the formation of free radicals [40], altering the properties of membrane lipids. Therefore, we settled on a pulsed sonication duration of 30 min.

### 3.3. The Effect of Chol Content

**Figure 4:**
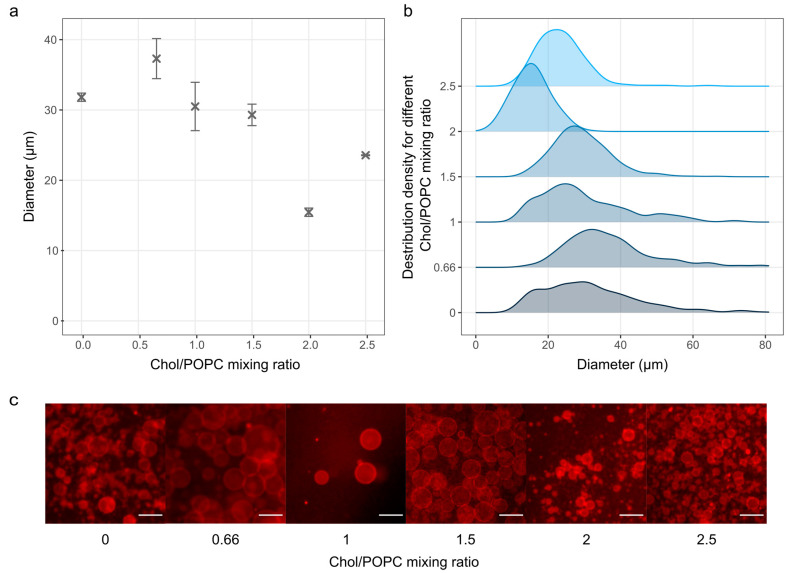
The effect of increasing the Chol content on the electroformation of GUVs. (**a**) Size of GUVs as a function of the different Chol mixing ratios. The points and bars represent mean values and standard errors. The mean values were calculated by averaging the mean diameters from three independent samples for each concentration. (**b**) Size distribution densities for different Chol contents. Each distribution density represents 300 vesicles (100 vesicles from each of the three samples). (**c**) Fluorescence microscopy images for each sample. The scale bar represents 50 µm.

After finding the optimal sonication parameters, we tested the effect of increasing the Chol concentration on GUV electroformation using our modified protocol (Figure 4). The results indicate a decrease in the average diameter of GUVs with increasing Chol/POPC ratio, with a size range similar to that observed in previous studies [12].

This trend could be a result of reduced membrane elasticity and increased rigidity when more Chol is incorporated, which hinders vesicle formation. The effect is especially pronounced when the Chol/POPC mixing ratio exceeds 1.5 (more than 60 mol% Chol in the mixture), where GUV membranes are expected to contain pure cholesterol bilayer domains (CBDs) [17]. An increase at a ratio of 2.5 (71.5 mol% Chol in the mixture) could be explained by Chol demixing due to the Chol/POPC ratio being higher than the Chol solubility limit of 66 mol% [41]. The demixing was also evident from a high number of Chol crystals observed during microscopy after the Chol/POPC ratio exceeded two. No Chol crystals were detected at the Chol/POPC mixing ratio lower than 2. At a ratio of 2, a negligible number of crystals were found, which was much lower than the number observed at the same ratio when using the traditional protocol. As expected, many crystals appeared on the surface after exceeding the Chol solubility limit at a Chol/POPC ratio of 2.5.

### 3.4. The Effect of Charged Lipids

**Figure 5:**
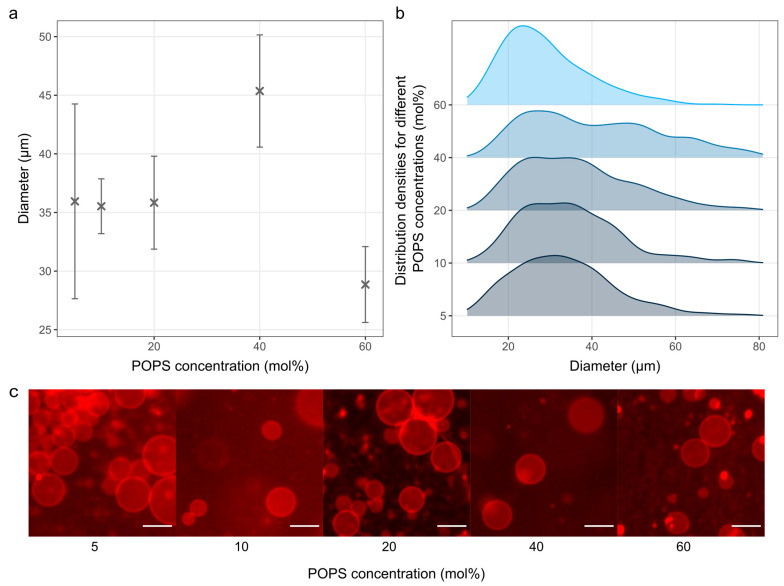
Electroformation from damp lipid films using different concentrations of POPS. (**a**) GUV size as a function of POPS concentrations. The points and bars represent mean values and standard errors. Mean values were calculated by averaging the mean diameters of three independent samples for each concentration. (**b**) Size distribution densities of GUVs for different POPS concentrations. Each distribution density represents 300 vesicles (100 vesicles from each of the three samples). (**c**) Fluorescence microscopy images for each sample. The scale bar represents 50 µm.

The effect of different concentrations of a negatively charged POPS lipid was also tested (Figure 5). 

We succeeded in producing GUVs from a ternary POPS/POPC/Chol mixture with a POPS concentration ranging from 5 to 60 mol%, and a fixed Chol concentration of 10 mol%. The number of vesicles produced at POPS concentrations higher than 60 mol% was negligible. 

Regarding the amount of POPS incorporated into the membrane, it has been shown that the incorporation of negatively charged lipids reduces membrane stability due to the reduction in membrane edge tension, which can lead to the complete collapse of the vesicle [42]. The results using POPC/POPG mixtures indicate high instability after 50 mol% of POPG [42], which is in line with the results presented here.

In comparable studies (using DOPS instead of POPS), an even lower amount of charged lipids was used for testing, namely about 30 mol% [23,43]. The average diameters of the obtained vesicles with such a composition were about 10 μm [43] and 20 μm [23], which is much smaller compared to our results with similar concentrations of charged lipids. 

It should also be noted that one of these studies performed a comprehensive optimization of the electrical parameters for each lipid composition [23], whereas we contented ourselves with the commonly used values. Additionally, in the aforementioned studies, the drop deposition method was used (with or without subsequent smearing) to deposit the lipid film. Although this approach is inherently less reproducible, it has the advantage of resulting in a lipid film with regions of varying thicknesses, ensuring that there is always a portion of the lipid film that has the optimal thickness for that lipid composition. Our protocol uses the spin-coating technique instead, which can lead to greater homogeneity of film thickness, but requires more optimization because film thickness varies greatly with different lipid compositions and lipid concentrations [8,11]. Consequently, even though the present results with POPS are already quite good, we expect even better outcomes once the fine-tuning of the electrical parameters and film thickness is performed.

### 3.5. The Effect of Using Saline Solutions

**Figure 6:**
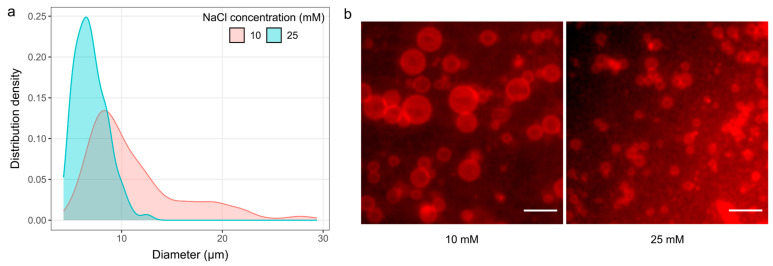
Electroformation from damp lipid films using saline solutions for the Chol/POPC mixture with a fixed Chol concentration of 10 mol%. (**a**) Size distribution of GUVs for different salt concentrations. Each distribution density represents 300 vesicles (100 vesicles from each of the three samples). (**b**) Fluorescence microscopy images for each sample. The scale bar represents 50 µm.

GUVs prepared from a Chol/POPC mixture with a fixed Chol concentration of 10 mol% can also be successfully produced even when saline solutions were used during electroformation (Figure 6). We selected a voltage of 4 V and a frequency of 500 Hz according to the literature [22]. The average GUV diameters and standard errors were 11.2 ± 2.6 µm and 7 ± 0.3 µm for NaCl concentrations of 10 and 25 mM, respectively.

Previous studies have shown that cleaning the electrodes with plasma [22] or applying preformed liposome suspensions during film deposition [43,44] can promote GUV electroformation under ionic conditions. Our modified protocol applies both of these modifications, and the results obtained are in agreement with those presented in their studies under similar conditions [22].

It is important to note that visibly wet lipid films were used for these experiments, as the duration of spin-coating was only 30 s. Since the amount of Chol in the mixture here was low, we could have dried the film even more, which would probably have given us even better results [10].

## 4. Conclusions

GUVs are extensively used in studies on membrane properties and are most commonly produced using the electroformation method. In this study, a modified protocol is described that addresses some of the shortcomings of the traditional method, offering a more efficient and reproducible approach for the preparation of GUVs, particularly for experiments requiring high Chol concentrations. In order to eliminate the dry lipid film phase and reduce Chol demixing, we used techniques that have been shown to be beneficial for GUV production under other conditions, such as when using charged lipids or ionic solutions.

Initially, the RSE method was used to avoid drying the lipid film and Chol demixing artifact. Sonication is a new method included in this protocol. We concluded that a sonication time of 30 min is sufficient to form LUVs. Replacing the extrusion step with sonication has further improved our protocol. Sonication is less time-consuming and results in a smaller amount of lipids used in the protocol. The extrusion of MLVs with a high Chol content, which results in a very large opposing force and makes the extrusion process difficult, was a problematic step in our previous protocol. Applying the sonication method instead of extrusion in our protocol makes the process shorter and easier to perform. We have also included plasma cleaning of the electrode, which has been shown to promote vesicle formation when using charged lipids or saline solution. To ensure methodological reproducibility, the solution of LUVs was applied to the electrode by spin-coating instead of drop deposition.

We tested our modified protocol using lipid mixtures with very high Chol concentrations, charged lipids (POPS), and saline solutions. Compared to other studies testing GUV electroformation under similar conditions, the results obtained were similar or better than the data found in the literature [22]. Furthermore, by bypassing the dry phase, our protocol should be more suitable for protein insertion into GUVs and further reduce the risk of protein denaturation [45,46]. 

In the future, we plan to test the performance of the new protocol using a larger number of lipid species and ionic solutions to further customize the protocol for the best possible outcomes. 

## Data Availability

The data presented in this study are available upon reasonable request from the corresponding author.

## References

[1] Rideau E., Dimova R., Schwille P., Wurm F.R., Landfester K. (2018). Liposomes and Polymersomes: A Comparative Review towards Cell Mimicking. Chem. Soc. Rev..

[2] Gudheti M.V., Mlodzianoski M., Hess S.T. (2007). Imaging and Shape Analysis of GUVs as Model Plasma Membranes: Effect of Trans DOPC on Membrane Properties. Biophys. J..

[3] Angelova M.I., Dimitrov D.S. (1986). Liposome Electroformation. Faraday Discuss. Chem. Soc..

[4] Angelova M., Dimitrov D.S. (1988). Angelova, M.; Dimitrov, D.S. A Mechanism of Liposome Electroformation. Trends in Colloid and Interface Science II.

[5] Veatch S.L. (2007). Electro-Formation and Fluorescence Microscopy of Giant Vesicles with Coexisting Liquid Phases. Methods Mol Biol..

[6] Morales-Penningston N.F., Wu J., Farkas E.R., Goh S.L., Konyakhina T.M., Zheng J.Y., Webb W.W., Feigenson G.W. (2010). GUV Preparation and Imaging: Minimizing Artifacts. Biochim. Biophys. Acta—Biomembr..

[7] Boban Z., Mardešić I., Subczynski W.K., Raguz M. (2021). Giant Unilamellar Vesicle Electroformation: What to Use, What to Avoid, and How to Quantify the Results. Membranes.

[8] Estes D.J., Mayer M. (2005). Electroformation of Giant Liposomes from Spin-Coated Films of Lipids. Colloids Surf. B Biointerfaces.

[9] Boban Z., Puljas A., Kovač D., Subczynski W.K., Raguz M. (2020). Effect of Electrical Parameters and Cholesterol Concentration on Giant Unilamellar Vesicles Electroformation. Cell Biochem. Biophys..

[10] Boban Z., Mardešić I., Jozić S.P., Šumanovac J., Subczynski W.K., Raguz M. (2023). Electroformation of Giant Unilamellar Vesicles from Damp Lipid Films Formed by Vesicle Fusion. Membranes.

[11] Boban Z., Mardešić I., Subczynski W.K., Jozić D., Raguz M. (2022). Optimization of Giant Unilamellar Vesicle Electroformation for Phosphatidylcholine/Sphingomyelin/Cholesterol Ternary Mixtures. Membranes.

[12] Mardešić I., Boban Z., Raguz M. (2024). Electroformation of Giant Unilamellar Vesicles from Damp Lipid Films with a Focus on Vesicles with High Cholesterol Content. Membranes.

[13] Politano T.J., Froude V.E., Jing B., Zhu Y. (2010). AC-Electric Field Dependent Electroformation of Giant Lipid Vesicles. Colloids Surf. B Biointerfaces.

[14] Billerit C., Jeffries G.D.M., Orwar O., Jesorka A. (2012). Formation of Giant Unilamellar Vesicles from Spin-Coated Lipid Films by Localized IR Heating. Soft Matter.

[15] Baykal-Caglar E., Hassan-Zadeh E., Saremi B., Huang J. (2012). Preparation of Giant Unilamellar Vesicles from Damp Lipid Film for Better Lipid Compositional Uniformity. Biochim. Biophys. Acta—Biomembr..

[16] Raguz M., Kumar S.N., Zareba M., Ilic N., Mainali L., Subczynski W.K. (2019). Confocal Microscopy Confirmed That in Phosphatidylcholine Giant Unilamellar Vesicles with Very High Cholesterol Content Pure Cholesterol Bilayer Domains Form. Cell Biochem. Biophys..

[17] Mainali L., Raguz M., Subczynski W.K. (2013). Formation of Cholesterol Bilayer Domains Precedes Formation of Cholesterol Crystals in Cholesterol/Dimyristoylphosphatidylcholine Membranes: EPR and DSC Studies. J. Phys. Chem. B.

[18] Buboltz J.T., Feigenson G.W. (1999). A Novel Strategy for the Preparation of Liposomes: Rapid Solvent Exchange. Biochim. Biophys. Acta—Biomembr..

[19] Buboltz J.T. (2009). A More Efficient Device for Preparing Model-Membrane Liposomes by the Rapid Solvent Exchange Method. Rev. Sci. Instrum..

[20] Mainali L., Raguz M., O’Brien W.J., Subczynski W.K. (2013). Properties of Membranes Derived from the Total Lipids Extracted from the Human Lens Cortex and Nucleus. Biochim. Biophys. Acta—Biomembr..

[21] Park S., Sut T.N., Ma G.J., Parikh A.N., Cho N.J. (2020). Crystallization of Cholesterol in Phospholipid Membranes Follows Ostwald’s Rule of Stages. J. Am. Chem. Soc..

[22] Li Q., Wang X., Ma S., Zhang Y., Han X. (2016). Electroformation of Giant Unilamellar Vesicles in Saline Solution. Colloids Surf. B Biointerfaces.

[23] Ghellab S.E., Mu W., Li Q., Han X. (2019). Prediction of the Size of Electroformed Giant Unilamellar Vesicle Using Response Surface Methodology. Biophys. Chem..

[24] Preston Mason R., Tulenko T.N., Jacob R.F. (2003). Direct Evidence for Cholesterol Crystalline Domains in Biological Membranes: Role in Human Pathobiology. Biochim. Biophys. Acta—Biomembr..

[25] Mainali L., O’Brien W.J., Subczynski W.K. (2019). Detection of Cholesterol Bilayer Domains in Intact Biological Membranes: Methodology Development and Its Application to Studies of Eye Lens Fiber Cell Plasma Membranes. Exp. Eye Res..

[26] Herold C., Chwastek G., Schwille P., Petrov E.P. (2012). Efficient Electroformation of Supergiant Unilamellar Vesicles Containing Cationic Lipids on ITO-Coated Electrodes. Langmuir.

[27] Schindelin J., Arganda-Carreras I., Frise E., Kaynig V., Longair M., Pietzsch T., Preibisch S., Rueden C., Saalfeld S., Schmid B. (2012). Fiji: An Open-Source Platform for Biological-Image Analysis. Nat. Methods.

[28] R Development Core Team (2008). R: A Language and Environment for Statistical Computing.

[29] Richter R.P., Bérat R., Brisson A.R. (2006). Formation of Solid-Supported Lipid Bilayers: An Integrated View. Langmuir.

[30] Brian A.A., McConnell H.M. (1984). Allogeneic Stimulation of Cytotoxic T Cells by Supported Planar Membranes. Proc. Natl. Acad. Sci. USA.

[31] Lind T.K., Cárdenas M., Wacklin H.P. (2014). Formation of Supported Lipid Bilayers by Vesicle Fusion: Effect of Deposition Temperature. Langmuir.

[32] Jackman J.A., Cho N.-J. (2020). Supported Lipid Bilayer Formation: Beyond Vesicle Fusion. Langmuir.

[33] Tero R. (2012). Substrate Effects on the Formation Process, Structure and Physicochemical Properties of Supported Lipid Bilayers. Materials.

[34] Reeves J.P., Dowben R.M. (1969). Formation and Properties of Thin-Walled Phospholipid Vesicles. J. Cell. Physiol..

[35] Darszon A., Vandenberg C.A., Schönfeld M., Ellisman M.H., Spitzer N.C., Montal M. (1980). Reassembly of Protein-Lipid Complexes into Large Bilayer Vesicles: Perspectives for Membrane Reconstitution. Proc. Natl. Acad. Sci. USA.

[36] Rodriguez N., Pincet F., Cribier S. (2005). Giant Vesicles Formed by Gentle Hydration and Electroformation: A Comparison by Fluorescence Microscopy. Colloids Surf. B Biointerfaces.

[37] Cho N.J., Hwang L.Y., Solandt J.J.R., Frank C.W. (2013). Comparison of Extruded and Sonicated Vesicles for Planar Bilayer Self-Assembly. Materials.

[38] Lapinski M.M., Castro-Forero A., Greiner A.J., Ofoli R.Y., Blanchard G.J. (2007). Comparison of Liposomes Formed by Sonication and Extrusion: Rotational and Translational Diffusion of an Embedded Chromophore. Langmuir.

[39] Woodbury D.J., Richardson E.S., Grigg A.W., Welling R.D., Knudson B.H. (2006). Reducing Liposome Size with Ultrasound: Bimodal Size Distributions. J. Liposome Res..

[40] Maulucci G., De Spirito M., Arcovito G., Boffi F., Castellano A.C., Briganti G. (2005). Particle Size Distribution in DMPC Vesicles Solutions Undergoing Different Sonication Times. Biophys. J..

[41] Huang J., Feigenson G.W. (1999). A Microscopic Interaction Model of Maximum Solubility of Cholesterol in Lipid Bilayers. Biophys. J..

[42] Lira R.B., Leomil F.S.C., Melo R.J., Riske K.A., Dimova R. (2021). To Close or to Collapse: The Role of Charges on Membrane Stability upon Pore Formation. Adv. Sci..

[43] Uzun H.D., Tiris Z., Czarnetzki M., López-Marqués R.L., Günther Pomorski T. (2024). Electroformation of Giant Unilamellar Vesicles from Large Liposomes. Eur. Phys. J. Spec. Top..

[44] Pott T., Bouvrais H., Méléard P. (2008). Giant Unilamellar Vesicle Formation under Physiologically Relevant Conditions. Chem. Phys. Lipids.

[45] Girard P., Pécréaux J., Lenoir G., Falson P., Rigaud J.L., Bassereau P. (2004). A New Method for the Reconstitution of Membrane Proteins into Giant Unilamellar Vesicles. Biophys. J..

[46] Witkowska A., Jablonski L., Jahn R. (2018). A Convenient Protocol for Generating Giant Unilamellar Vesicles Containing SNARE Proteins Using Electroformation. Sci. Rep..

